# β-hydroxybutyrate enhances bovine neutrophil adhesion by inhibiting autophagy

**DOI:** 10.3389/fimmu.2022.1096813

**Published:** 2023-01-11

**Authors:** Jiyuan He, Kexin Wang, Mingchao Liu, Wen Zeng, Dong Li, Zolzaya Majigsuren, Tugsjargal Batbaatar, Yunfei Li, Siyuan Liu, Xiliang Du, Lin Lei, Yuxiang Song, Guowen Liu

**Affiliations:** ^1^ State Key Laboratory for Zoonotic Diseases, Key Laboratory of Zoonosis Research, Ministry of Education, College of Veterinary Medicine, Jilin University, Changchun, Jilin, China; ^2^ College of Veterinary Medicine, Hebei Agricultural University, Baoding, Hebei, China; ^3^ Institute of Veterinary Medicine, Mongolian University of Life Sciences, Ulaanbaatar, Mongolia; ^4^ State Central Veterinary Laboratory, General Authority for Veterinary Services, Ministry of Food And Agriculture, Ulaanbaatar, Mongolia

**Keywords:** dairy cows, ketosis, β-hydroxybutyrate, neutrophils, adhesion

## Abstract

**Introduction:**

Subclinical ketosis (SCK) in dairy cows, a common metabolic disorder during the perinatal period, is accompanied by systemic inflammation and a high concentration of blood β-hydroxybutyrate (BHB). BHB induced adhesion of neutrophils may play a crucial role in the development of systemic inflammation in SCK cows. Autophagy, an intracellular degradation system, regulates the recycling of membrane adhesion molecules and may be involved in BHB regulating adhesion and pro-inflammatory activation of bovine neutrophils. Thus, the objective of this study was to determine the relationship between BHB, autophagy, and neutrophil adhesion.

**Results and discussion:**

Here, elevated abundance of serum amyloid A, haptoglobin, C-reactive protein, interleukin-1β, interleukin-6, and tumor necrosis factor-α were found in SCK cows, and all these pro-inflammatory factors had a strong positive correlation with serum BHB. After BHB treatment, the number of adherent neutrophils and the adhesion associated protein abundance of both total and membrane CD11a, CD11b, and CD18 was greater, confirming that BHB promoted the adhesion of bovine neutrophils. However, the mRNA abundance of *ITGAL* (CD11a), *ITGAM* (CD11b), and *ITGB2* (CD18) did not show a significant difference, suggesting that the degradation of adhesion molecules may be impaired. Transmission electron microscopy revealed a decreased number of autophagosomes and a decrease in mRNA abundance of *SQSTM1* (p62) and *MAP1LC3B* (LC3) after BHB treatment. In parallel, protein abundance of p62 increased while the ratio of protein LC3 II to LC3 I decreased after BHB treatment, indicating that BHB inhibits autophagy of bovine neutrophils. To confirm the regulatory role of autophagy in BHB promoting neutrophil adhesion, we used an autophagy activator rapamycin (RAPA). Data showed that RAPA relieved the inhibitory effect on autophagy and the promotive effect on cell adhesion induced by BHB. Importantly, BHB inhibited the colocalization of LC3 and CD11b, which was relieved by RAPA, further confirming the regulatory role of autophagy in the recycling of the above adhesion molecules. Furthermore, BHB treatment increased the mRNA abundance and the release of pro-inflammatory factors *IL-1B*, *IL-6*, and *TNF* of bovine neutrophils, and these effects were attenuated by RAPA. Overall, the present study revealed that BHB promotes the adhesion of bovine neutrophils by inhibiting autophagy.

## Introduction

Due to the increase in energy demand and the decrease in dry matter intake, dairy cows were subjected to a period of negative energy balance (NEB) during the transition period. NEB intensifies fat mobilization and increases the concentration of non-esterified fatty acid (NEFA) in blood (1). NEFAs are transported into the liver and involved in β-oxidation in the hepatocytes to generate more ATP to relieve the NEB, while excess NEFAs could not be completely oxidized and metabolized into ketones, such as β-hydroxybutyrate (BHB). Subclinical ketosis (SCK) is defined as an excess of circulating BHB (1.2 m*M* -3 m*M*) without clinical signs (2). The incidence rate of SCK can be as high as 36.6% in some herds, far higher than 2% to 15% in clinical ketosis (3). SCK cows experience systemic inflammation, which is characterized by enhanced blood levels of pro-inflammatory cytokines and positive acute-phase proteins (4). The systemic inflammation during SCK is closely related to secondary and concurrent inflammatory diseases, such as mastitis, endometritis, and laminitis (3), and the reduction in production performance and welfare (2). The underlying mechanism of systemic inflammation in SCK cows is still unclear.

BHB, the most abundant ketone body in blood, is not only an energy precursor but an immuno-modulating molecule. Some previous reports have shown that BHB can significantly dampen central nervous system inflammation and exert protective effects in mice and humans (5, 6), whereas, the expression of pro-inflammatory factors significantly upregulated in BHB exposure hepatocytes (7). Therefore, the inflammation‐regulatory effects of BHB seem to be context or cell type dependent. Additionally, in ruminants, the pathological concentration of BHB can cause cellular dysfunction such as inhibiting apoptosis of neutrophils, liver damage, and promoting lipolysis of adipocytes (8–10). Thus, the effect of BHB on the immune cells of dairy cows needs to be further studied.

Neutrophils are the first line against the invasion of pathogenic microbes; however, abnormal excessive neutrophil recruitment may result in chronic inflammation and tissue injury. Neutrophils adhere to endothelium or extracellular matrix in a coordinated and reversible manner to start up the chemotaxis process and pro-inflammatory activation. Firm adhesion is mediated by the interaction of integrin on neutrophils with receptors on endothelial cells before recruiting to the local inflammatory sites (11). Neutrophils express high levels of the β2 integrins which are heterodimer composed of a variable α subunit including lymphocyte function-associated antigen-1 (LFA-1, also known as CD11a), macrophage 1 antigen-1 (Mac-1, also known as CD11b), and a constant β subunit (also known as CD18) (12). Synthesis, transportation, membrane location, and intracellular degradation of adhesive molecules are in a state of dynamic circulation (13). Additionally, through an intracellular-signaling process (also known as inside-out signaling), the transformation of integrins to the active conformation can be accelerated, thereby ultimately modulating their affinity for ligands (14). In mice, enhancement of adhesion between neutrophils and vascular endothelial cells causes systemic inflammation and injury of blood vessels and peripheral organs (15). In humans and rats, hyperketonemia (elevated blood ketones) can increase the expression of CD11a in monocytes and macrophages and ICAM I in endothelial cells to promote adhesion between them, potentiating infiltration of monocytes and macrophages into the aorta and liver (16). However, the effect of BHB on the adhesion of bovine neutrophils and the underlying mechanisms are still not clarified.

In both immune and non-immunized cells, autophagy was shown involved in adhesion regulation through balancing the endocytosis, circulation, and degradation of integrins (17, 18). The number of infiltrated neutrophils in microvascular venules increased significantly in Atg5^-/-^ mice (19).

In SCK cows, blood neutrophils are exposed to high concentrations of BHB. Given the potential role of BHB on the adhesion and the involvement of autophagy in regulating cell adhesion, we speculated that BHB may enhance neutrophil adhesion through inhibiting autophagy in SCK cows. The objective of this study was to determine the effect of BHB on the adhesion of bovine neutrophils and the underlying mechanisms.

## Materials and methods

### Animals and sampling

The protocol was approved by the Ethics Committee on the Use and Care of Animals of Jilin University (No. SY202012016). In the current study, experimental animals received humane care according to the principles and guidelines of the “Guidelines for the Care and Use of Agricultural Animals, 3rd ed” (available from FASS Inc., 1800 S. Oak St., Suite 100, Champaign, IL 61820, USA). In general, ketosis cows can be initially screened by ketone powder testing for ketosis in the milk, and the final diagnosis is confirmed by testing the serum concentration of ketosis. Based on a nitroprusside test for milk ketone bodies, we screened out 23 suspected SCK and 30 control cows with similar parity (median = 3, range = 2 to 4) and days in milk (DIM) (median = 6 d, range = 3 to 15 d) from 100 lactating Holstein cows in a dairy farm located in Changchun City, Jilin Province, China. All screened cows received a routine physical examination to ensure the absence of clinical symptoms and comorbidities. Milk samples were also collected for somatic cell count (SCC) using the Delaval Cell Counter (DeLaval International AB), bacteriological examination by inoculation and culture on bacterial medium, and identification of antimicrobial residues using the SNAP tetracycline test kit, the SNAP β-lactam test kit, and the SNAP gentamicin test kit (Idexx Laboratories). Both bacteriological and antimicrobial residue examinations were negative in the milk of selected cows. Additionally, it has been demonstrated that SCC ≤ 200,000 cells/ml could be considered a threshold for mastitis (20). Cows were divided into two groups according to the concentration of serum β-hydroxybutyrate (BHB): healthy cows (control group, n = 15) with BHB < 0.6 m*M* and SCK cows (SCK group, n = 15) with 1.2 < BHB < 3 m*M*. All selected cows were housed in a climate-controlled barn with individual tie stalls to reduce environmental interference. The selected cows were milked twice daily at 08:00 h and 15:30 h. Cows had ad libitum access to the same diet (21) that was offered twice daily (09:00 and 16:30 h) and freshwater was supplied continuously.

Blood samples were collected from the jugular vein at 06:30-07:30 h before feeding for 3 consecutive days. To obtain serum, blood samples were kept for 30 min at room temperature then centrifuged at 4°C for 3,000 × g for 15 min and stored at -80 °C immediately until use. Serum glucose (GLU) and BHB were detected using commercially available kits (GLU, Cat. No. GL3815; BHB, Cat. No. RB1008; Randox Laboratories, Crumlin, County Antrim, UK) using a Hitachi 7170 autoanalyzer (Hitachi; Tokyo; Japan). Serum metabolic indices for the selected control and SCK cows are reported in **Table 1**.

**Table 1:** Basic description of the control and SCK cows.

	Control cows	SCK cows	
Item	(n = 15)	(n = 15)	*P*-value
Serum BHB (m*M*)	0.39 ± 0.43	1.89 ± 0.12	< 0.01
Serum Glucose (m*M*)	4.19 ± 0.13	3.35 ± 0.22	< 0.01
Serum SAA (µg/mL)	42.23 ± 4.98	120.07 ± 9.47	< 0.01
Serum HP (µg/mL)	128.65 ± 13.01	443.45 ± 53.49	< 0.01
Serum CRP (µg/mL)	20.34 ± 2.65	94.75 ± 3.65	< 0.01
Serum TNF-α (pg/mL)	80.45 ± 6.52	401.02 ± 28.33	< 0.01
Serum IL-1β (pg/mL)	48.97 ± 9.22	79.88± 6.78	< 0.01
Serum IL-6 (pg/mL)	13.44 ± 2.21	49.93 ± 5.98	< 0.01

The concentrations of BHB, Glucose, SAA, HP, CPR, TNF-α, IL-1β, and IL-6 in the serum of control and subclinical ketosis cows have been shown. Values are shown as mean ± SEM. P < 0.05 was considered significant and P < 0.01 highly significant. DMI, dry matter intake; BHB, β-hydroxybutyrate; SAA, serum amyloid A protein; HP, haptoglobin; CRP, C-reactive protein; IL, Interleukin; TNF-α, Tumor necrosis factor-α.

### Detection of serum inflammatory factors

Concentrations of serum haptoglobin (HP), serum amyloid A (SAA), and C-reactive protein (CRP) were determined with bovine specific ELISA kits (HP, LS-F13229; SAA, LS-F12552, LifeSpan BioSciences Inc., Seattle, Washington, USA; CRP, CSB-E08577b, GUSABIO, China) according to manufacturer’s instructions. Kit sensitivity of HP, SAA, and CRP was 7.8 ng/mL, 3.12 ng/mL, and 1.25 ng/mL, respectively. Intra-/inter-assay coefficient of variation (CV) for HP, SAA, and CRP was less than 6.5%/8.8%, 10%/12%, and 8%/10%, respectively. Concentrations of interleukin-1 beta (IL-1β), interleukin-6 (IL-6), and tumor necrosis factor-α (TNF-α) were measured with bovine specific ELISA kits (IL-1β, LS-F7588; IL-6, LS-F9752; TNF-α, LS-F5014; LifeSpan BioSciences Inc., Seattle, Washington, USA) according to the manufacturer’s instructions. Kit sensitivity of IL-1β, IL-6, and TNF-α was 6.5 pg/mL, 0.46 pg/mL, and 3.1 pg/mL, respectively. Intra- and inter-assay CV for IL-1β, IL-6, and TNF-α were all less than 10%. Correlations between the content of serum HP, SAA, CRP, IL-1β, IL-6, TNF-α, and BHB in serum were analyzed with the Spearman method. All samples including standards were tested in triplicate.

### Cell adhesion assay

For adhesion assays, the 96-well plates were pre-coated with Collagen I (50 µg/mL, Cat. No. C8062, Solarbio Science and Technology Co. Ltd.; Beijing; China) overnight. After isolation, neutrophils were adjusted to 1×10^5^ cells/mL. The 100 μL cell suspension was added to a 96-well plate and placed in a 37°C and 5% CO_2_ incubator for 2 hours. The non-adherent cells were removed by washing 3 times with PBS solution. The number of remaining cells was counted under a microscope (Fluoview FV1200; OLYMPUS; Tokyo; Japan). The number of adherent neutrophils was calculated by Image J (Media Cybernetics Inc., Bethesda, Maryland, USA). For each sample, at least 5 watching zones were randomly selected for evaluation.

### Isolation, culture, and treatment of neutrophils

Blood was collected by jugular puncture and kept in a sterile EDTA vacutainer tube (YA1393, Solarbio Science and Technology Co. Ltd., Beijing, China) at room temperature until transported to the laboratory, within 30 min after collection. Neutrophils were isolated by the commercial bovine peripheral blood neutrophils isolation kit following the manufacturer’s protocol (Cat. No. P9400; Solarbio Science and Technology Co. Ltd.; Beijing; China). In brief, neutrophils were separated based on the density gradient centrifugation method. The whole process was carried out in a sterile room at room temperature. Sixteen mL of liquid A and 8 mL of liquid B were slowly added to the 50 mL centrifuge tube. 15 mL of fresh bovine blood was added to the upper layer of liquid B in the same way and centrifuged for 30 min at 800 × g. After centrifugation, the second layer of white turbidity layers was sucked into another sterile centrifuge tube and mixed with 20 mL of cleaning solution. After centrifugation, the supernatant was discarded and 10 mL of red cell lysate was added. neutrophils were obtained through 800 × g for 10 min. The purity of isolated neutrophils was determined by double staining of CH138A (22) and CD11b, and flow cytometry showed that the purity was 97.8%. The cells were diluted with RPMI-1640 (Cat. No. SH30809.01; HyClone; Logan, UT, United States) containing 10% fetal bovine serum (FBS; Cat. No. FB15015; Hyclone Laboratories). The cell density was adjusted to 2×10^6^ cells/mL to conduct the following experiment.

BHB (Cat. No. 55397; Sigma‐Aldrich) powder was dissolved in PBS, and stored under -80 °C conditions after sterilizing by filtration. The BHB concentrations used in this study were selected according to normal and pathological hematological criteria of cows with or without ketosis. Accordingly, neutrophils were maintained in RPMI-1640 and treated with 1.6 m*M* (subclinical level) for 0, 1, 2, 4, and 6 h. Two hours were selected as the treatment time for subsequent experiments according to an obvious effect on neutrophil adhesion. Neutrophils were then treated with 0, 0.8 (normal level), 1.6 (subclinical level), and 3.2 m*M* (clinical level) for 2 h, and 1.6 m*M* BHB treatment had an obvious effect on neutrophil adhesion. Thus, 1.6 m*M* was selected as the treatment concentration for subsequent experiments. To verify the regulatory role of autophagy signaling, the autophagy activator rapamycin (100 µ*M*, Cat. No. AY-22989; Selleck; Texas; USA) was preincubated for 0.5 h before BHB treatment, respectively.

### Protein extraction and Western blotting

Membrane protein of neutrophils was extracted using a commercial protein extraction kit (Cat. No. P0033; Beyotime; Shanghai; China). The total protein was extracted from neutrophils using a commercial Protein Extraction Kit (C510003, SANGON Biotechnology Co. Ltd., Shanghai, China) according to the manufacturer’s instructions. Protein concentration was measured *via* a BCA method (P1511, Applygen Technologies, Beijing, China). A total of 30 μg of protein from each sample was separated by 12% SDS PAGE. The target protein on the gel was transferred to 0.45-µm polyvinylidene difluoride membranes (PVDF, YA1701, Solarbio Science and Technology Co. Ltd., Beijing, China). The PVDF membranes were blocked with Tris-buffered saline solution with 0.1% Tween-20 (T8220, Solarbio Science and Technology Co. Ltd., Beijing, China) in 3% BSA (A8850, Solarbio Science and Technology Co. Ltd., Beijing, China) for 2 h at room temperature. After electrophoresis, transfer, and blocking, immunoblots were performed using the primary antibody of β-actin (1:2000; ab8226, Abcam, Cambridge, UK), CD11a (1:1000; bs-20370R, Bioss, Beijing, China), CD11b (1:1000; NB110-89474F, Novus Biologicals, Littleton, Colorado, USA), CD18 (1:1000; ab62817, Abcam, Cambridge, UK), and Na^+/^K^+^ ATPase (1:800; bs-23413R, Bioss, Beijing, China), p62 (1:1000; PA5-27247, Thermo Fisher Scientific, Massachusetts, USA), and LC3 (1:1000; ab128025, Abcam, Cambridge, UK) at 4°C overnight, respectively. Subsequently, the PVDF membranes were washed 3 times with Tris-buffered saline solution with 0.1% Tween-20 (TBST) and incubated with horseradish peroxidase-conjugated anti-mouse (1:5000; SA00001-1, ProteinTech Group, Inc., Wuhan, China) or anti-rabbit secondary antibodies (1:5000; SA00001-2, ProteinTech Group Inc., Wuhan, China) for 45 min at room temperature. After washing the PVDF membranes another 3 times with TBST, immunoreactive bands were visualized by enhanced chemiluminescence solution (ECL, WBKLS0500, Millipore, Bedford, Massachusetts, USA). β-actin was used as a reference protein for total proteins, Na^+^/K^+^ ATPase was used as a reference protein for membrane proteins. Lastly, all bands were imaged using a Protein Simple Imager (ProteinSimple, Santa Clara, CA, USA). All protein bands were quantified using Image-Pro Plus 6.0 (Media Cybernetics Inc., Bethesda, Maryland, USA). Western blotting was run in triplicate for each experimental group.

### Transmission electron microscope

The number and the ultrastructural characteristics of autophagosomes in cells were evaluated by Transmission Electron Microscope (H-7650 electron microscope, Hitachi, Tokyo, Japan). Neutrophils isolated from SCK and control dairy cows were centrifuged at 1000 × g, 4°C for 10 min After being washed with PBS, 4% glutaraldehyde was slowly added along the tube wall to fix the cell mass. It was placed upright in the refrigerator at 4°C overnight and then fixed with 1% osmium tetroxide at 4°C for 2 h. After washing again, cells were dehydrated in a series of ethanol solutions (70%, 80%, 90%, 100%, 100%, and 100%) and permeated in Spurr’s resin. Cells were sliced with an ultra-thin microtome of about 70 nm and dyed with 2% uranyl acetate for 10 min. Cell sections were then dyed with 0.3% lead citrate for 10 minutes and rinsed 3 times with distilled water. After thoroughly air-dried, the sections were observed under an H-7650 transmission electron microscope (Hitachi, Ibaraki Prefecture, Japan). The number of autophagosomes was counted from at least 5 random cell sections in each sample and expressed as the average number of each cell section.

### Quantitative reverse-transcription PCR assay

Ribonucleic acid was extracted from cell samples using Trizol (Cat. No. 15596026; Invitrogen; Carlsbad; CA), according to the manufacturer’s instructions, and dissolved with 20-30 µL of RNase-free water. Use a k5500 microspectrophotometer (Beijing Kaiao Technology Development Ltd.; Beijing; China) to determine the concentration and quality of RNA. One μg RNA from each sample was reverse-transcribed to cDNA using a PrimeScript Reverse Transcriptase Kit (Cat. No. 6110B; TaKaRa Biotechnology Co. Ltd.; Tokyo; Japan). The mRNA abundance was detected using an SYBR green plus reagent kit (Cat. No. 4913850001; Roche; Norwalk; CT) with the 7500 Real-Time PCR System (Applied Biosystems Inc.; Waltham; MA). Relative gene expression was normalized with *β-Actin* and *tyrosine 3-monooxygenase/tryptophan 5-monooxygenase activation protein zeta (YWHAZ)*. The PCR reactions were performed in triplicate for each of the 3 individual cell preps and determined by the 2^−ΔΔCT^ method (23). Primers of target genes, as shown in **Supplement Table 1**, were designed with Primer Express software (Applied Biosystems Inc.).

### Immunofluorescence assay

After treatment, neutrophils were harvested and washed twice with ice-cold PBS and fixed with 4% paraformaldehyde for 30 min at room temperature. After incubation with proteinase K for 1 min, neutrophils were permeabilized with Triton X-100 (0.1%) for 30 min. Subsequently, neutrophils were blocked with 5% goat serum for 1 h followed by incubation with the specific primary antibodies overnight at 4 °C. After washing with PBS 3 times, neutrophils were then incubated with the secondary antibody for 1 h at room temperature. Then, samples were washed with PBS 3 times and incubated with DAPI (Cat. No. D1306; Thermo Fisher Scientific) for 10 min. Fluorescence was observed using a Confocal Microscope (Fluoview FV1200; OLYMPUS; Tokyo; Japan). Co-localization analysis was done using Image J (Media Cybernetics; Rockville; Maryland). In brief, we used Coloc 2 co-localization plugin to analyze Pearson’s correlation coefficients of CD11b and LC3. The experiment was repeated 3 times, and at least 5 watching zones per sample were selected for evaluation.

### Statistical analysis

The one-way ANOVA or the independent-samples t-test was used when there was only one treatment factor. The two-way ANOVA was used when there were two treatment factors (BHB and rapamycin), including a Bonferroni *post-hoc* analysis when significant interaction occurred. All analyses were performed using SPSS (Statistical Package for the Social Sciences) 19.0 software (IBM). Correlation analysis was performed using the Spearman method. Weak, moderate, and strong correlations were defined as correlation coefficients (*R*) of 0 to 0.39, 0.40 to 0.59, and 0.6 to 1.0, respectively. *P* < 0.05 was considered significant and *P* < 0.01 was markedly significant.

## Results

### Systemic inflammation of SCK cows

Compared with the control group, SCK cows had a higher concentration of serum BHB (*P* < 0.01) and lower glucose (*P* < 0.01, **Table 1**). SCK cows had a greater (*P* < 0.01) concentration of positive acute phase proteins including HP, SAA, and CRP, as well as a greater (*P* < 0.01) concentration of pro-inflammatory cytokines including IL-1β, IL-6, and TNF-α in the serum (**Table 1**). These data suggest the development of SCK may be intimately tied to inflammation. Importantly, serum concentration of the above inflammatory factors had a strong positive correlation with that of BHB, respectively (**Table 2**).

**Table 2:** Correlation analysis between concentrations of pro-inflammatory factors and BHB in serum.

	Pro-inflammatory factors	HP	SAA	CRP	IL-1β	IL-6	TNF-α
BHB	*R*-value	0.8005	0.7986	0.8523	0.8772	0.9105	0.8012
	*P*-value	< 0.01	< 0.01	< 0.01	< 0.01	< 0.01	< 0.01

Weak, moderate, and strong correlations were defined as correlation coefficients (R) of 0 to 0.39, 0.40 to 0.59, and 0.6 to 1.0, respectively. P < 0.05 was considered significant and P < 0.01 highly significant. HP, Haptoglobin; SAA, Serum amyloid A; CRP, C-reactive protein; IL, Interleukin; TNF-α, Tumor necrosis factor-α; BHB, β-hydroxybutyrate.

### BHB enhances the adhesion of bovine neutrophils *in vitro*

**Figure 1:**
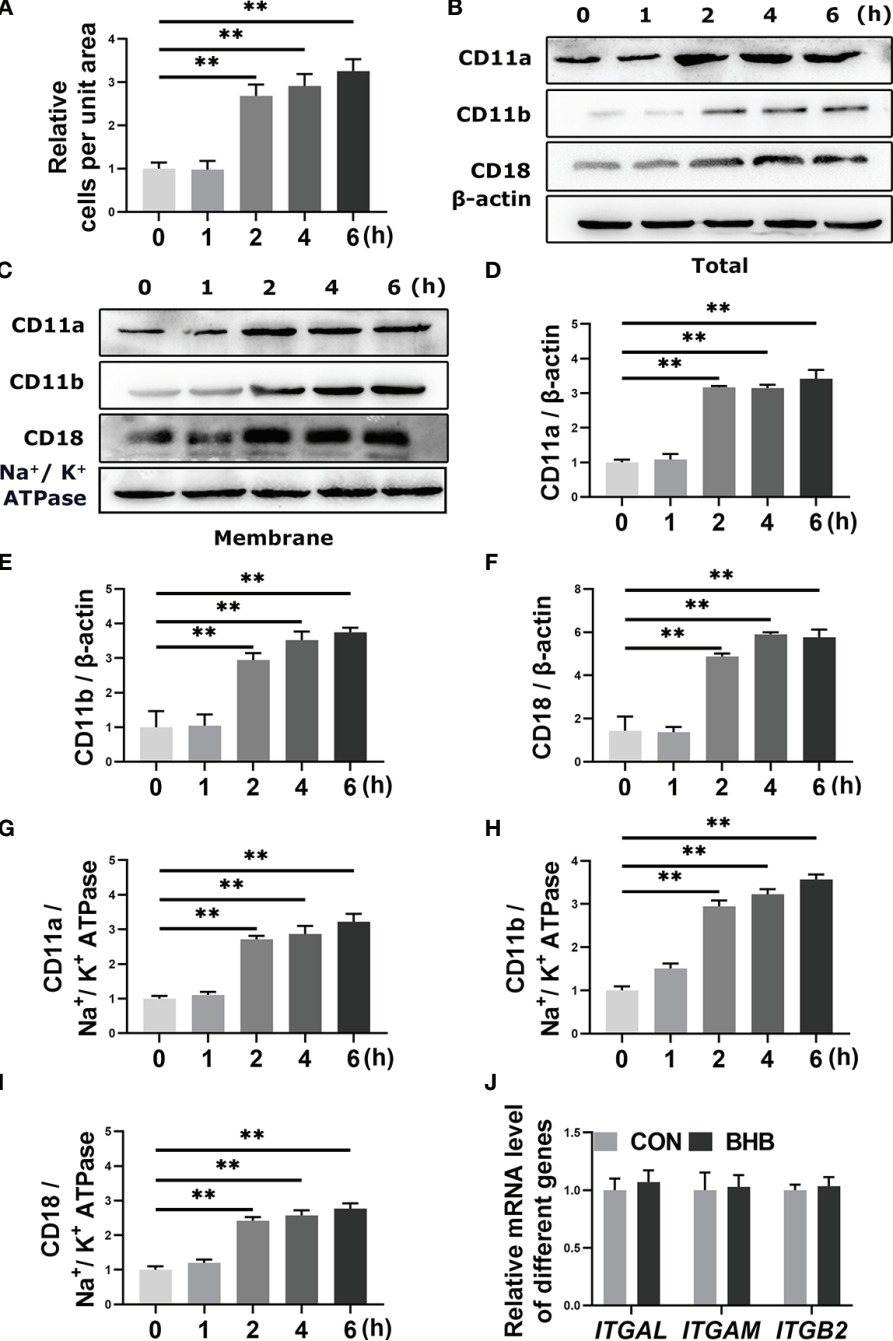
Effect of BHB with different stimulated times on the adhesion of bovine neutrophils. Neutrophils were treated with 1.6 m*M* BHB for 0, 1, 2, 4, and 6 h, respectively. **(A)** Quantitation of the number of adherent neutrophils. **(B-I)** Protein abundance of membrane and total CD11a, CD11b, and CD18. Representative blots in both groups were shown in B-C. The quantification was shown in D-I. **(J)** Neutrophils were treated with 1.6 m*M* BHB for 2h. mRNA abundance of *ITGAL*, *ITGAM*, and *ITGB2.* For WB experiments, β-actin was used to normalize the total protein abundance, while Na^+/^K^+^ ATPase was used to normalize membrane protein abundance; for qRT-PCR experiments, *β-Actin* and *YWHAZ* were used to normalize mRNA abundance. Data were expressed as the mean ± SEM. Data were analyzed with a one-way ANOVA with subsequent Bonferroni correction or independent-samples t-tests. **P < 0.01 as statistically highly significant. BHB, β-hydroxybutyrate; CD11b, integrin alpha-M precursor; CD11a, integrin alpha-L precursor; CD18, integrin beta-2 precursor; *ITGAL*, *integrin subunit alpha L; ITGAM*, *integrin subunit alpha M; ITGB2*, *integrin subunit beta 2; YWHAZ*, *tyrosine 3-monooxygenase/tryptophan 5-monooxygenase activation protein zeta*; SEM, standard error of the mean.

Bovine neutrophils were treated with 1.6 m*M* BHB for 0, 1, 2, 4, or 6 h *in vitro*. After 2 h of BHB treatment, the number of adherent cells increased significantly (*P* < 0.01, **Figure 1A**), and the total and membrane protein abundance of CD11a, CD11b, and CD18 increased significantly (*P* < 0.01, **Figures 1B-I**). It indicated that BHB can play the role in the enhancement of neutrophil adhesion, and 2 h BHB stimulation was chosen as the subsequent experimental condition. However, mRNA abundance of them was not significantly affected by BHB treatment (1.6 m*M*, 2 h) (**Figure 1J**), suggesting that the degradation of adhesion molecules is impaired.

**Figure 2:**
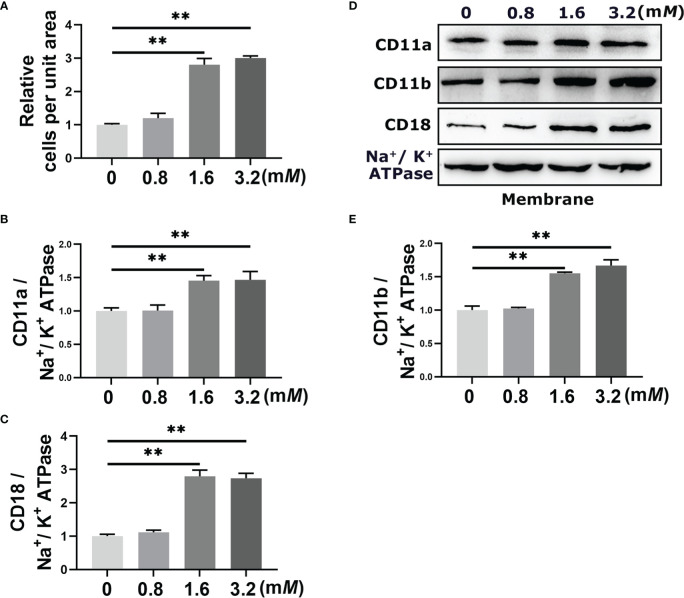
Effect of BHB with different stimulated concentrations on the adhesion of bovine neutrophils. Neutrophils were treated with 0 m*M*, 0.8 m*M*, 1.6 m*M*, and 3,2 m*M* BHB for 2 h, respectively. **(A)** Quantitation of the number of adherent neutrophils. **(B-E)** Protein abundance of membrane CD11a, CD11b, and CD18. Representative blots in both groups were shown in **(B)** The quantification was shown in C-E. For WB experiments, Na^+^/K^+^ ATPase was used to normalize membrane protein abundance. Data were expressed as the mean ± SEM. Data were analyzed with a one-way ANOVA with subsequent Bonferroni correction. **P < 0.01 as statistically highly significant. BHB, β-hydroxybutyrate; CD11b, integrin alpha-M precursor; CD11a, integrin alpha-L precursor; CD18, integrin beta-2 precursor; SEM, standard error of the mean.

The results of the adherent experiment showed that the number of adherent cells increased significantly when neutrophil was stimulated with the 1.6 m*M* and 3.2 m*M* BHB (*P* < 0.01, **Figure 2A**). There was no significant difference between the 1.6 m*M* and 3.2 m*M* BHB treatment groups, so 1.6 m*M* BHB was chosen as the stimulated concentration for the subsequent experiment. In terms of the western blot results, membrane protein abundance of CD11a, CD11b, and CD18 increased significantly after 1.6 m*M* BHB treatment (*P* < 0.01, **Figures 2B-E**).

### BHB impairs autophagy in bovine neutrophils

**Figure 3:**
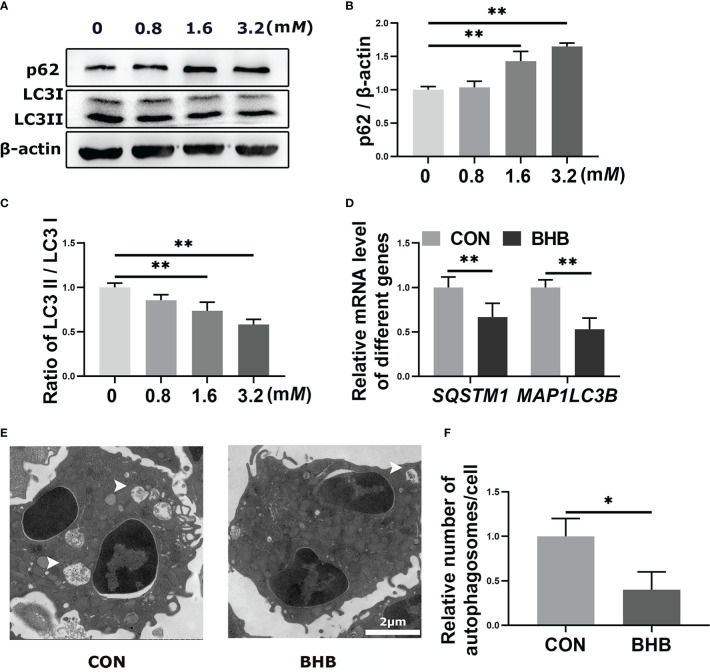
Effect of BHB on autophagy of bovine neutrophils. Neutrophils were treated with 0, 0.8, 1.6, and 3.2 m*M* BHB for 2 h, respectively. **(A-C)** Protein abundance of p62 and LC3. Representative blots in both groups were shown in A. The quantification was shown in B-C. Neutrophils were treated with 1.6 m*M* BHB for 2 h **(D)** mRNA abundance of *SQSTM1* and *MAP1LC3B*. **(E)** Representative TEM images showing the autophagosomes in neutrophils. The autophagosomes were labeled by arrowheads. Magnification, 2000× and Scale bars = 2 μm. **(F)** Relative average number of autophagosomes per cell. Autophagosomes were counted from at least 5 random complete views of cells in each sample and expressed as the number of autophagosomes per cell. For WB experiments, β-actin was used to normalize the total protein abundance. For qRT-PCR experiments, *β-Actin* and *YWHAZ* were used to normalize mRNA abundance. Data were expressed as the mean ± SEM. Data were analyzed with a one-way ANOVA with subsequent Bonferroni correction or independent-samples t-tests. **P < 0.01 as statistically highly significant. BHB, β-hydroxybutyrate; p62, sequestosome-1; LC3, microtubule associated proteins 1A/1B light chain 3; *SQSTM1*, *sequestosome-1*; *MAP1LC3B*, *microtubule associated protein 1 light chain 3 beta*; *YWHAZ*, *tyrosine 3-monooxygenase/tryptophan 5-monooxygenase activation protein zeta*; SEM, standard error of the mean.

To confirm the regulatory role of BHB in autophagy, bovine neutrophils were treated with BHB (0, 0.8, 1.6, or 3.2 m*M*) for 2 h *in vitro*. The protein abundance of p62 increased while the ratio of LC3 II to LC3 I decreased after treatment with a pathological concentration of BHB (1.6 m*M* and 3.2 m*M*) (*P* < 0.01, **Figures 3A-C**). Consistent with this, the pathological concentration of BHB decreased the mRNA abundance of *SQSTM1* and *MAP1LC3B* (*P* < 0.01, **Figure 3D**). Transmission electron microscopy revealed a lower average number of autophagosomes after BHB treatment (1.6 m*M*, 2 h) (*P =* 0.02, **Figures 3E, F**), indicating that BHB can inhibit neutrophil autophagy in cows with SCK.

### BHB enhances the adhesion of bovine neutrophils by inhibiting autophagy

**Figure 4:**
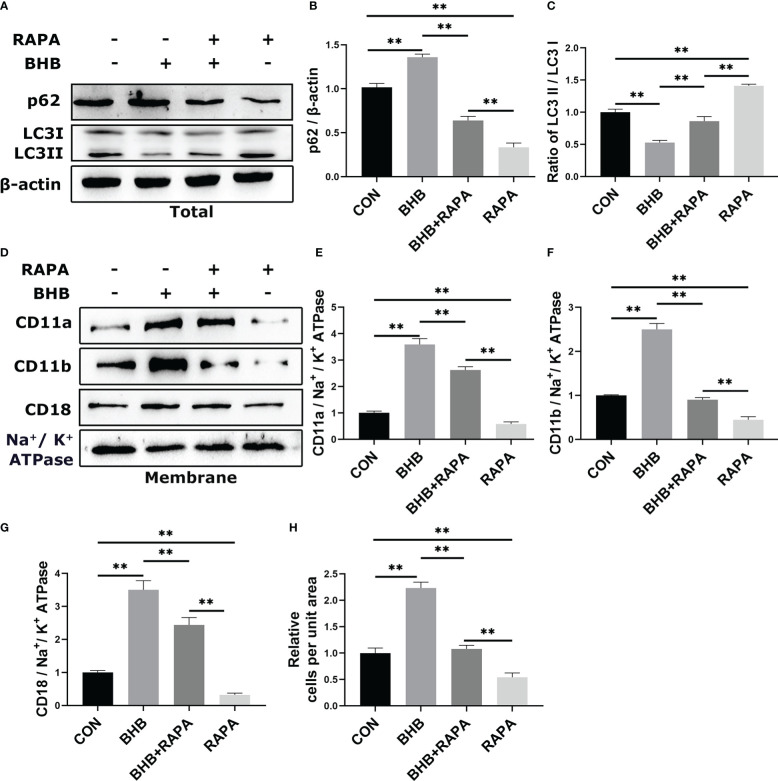
Role of autophagy in BHB promoting adhesion of bovine neutrophils. Rapamycin (100 n*M*, 2 h) was used to activate autophagy. **(A-C)** Protein abundance of p62 and LC3. Representative blots in both groups were shown in A. The quantification was shown in B-C. **(D-G)** Protein abundance of membrane CD11a, CD11b, CD18. Representative blots in both groups were shown in D. The quantification was shown in E-G. **(H)** Quantitation of the number of adherent neutrophils. For WB experiments, β-actin was used to normalize the total protein abundance, while Na^+/^K^+^ ATPase was used to normalize membrane protein abundance. Data were expressed as the mean ± SEM. A two-way analysis of variance (ANOVA) was performed to analyze differences between BHB and RARA, including a Bonferroni *post-hoc* analysis. Two-way ANOVA showed a significant difference in BHB × RAPA interaction (*P* < 0.01), including a Bonferroni *post-hoc* analysis. **P < 0.01 as statistically highly significant. CON, control; BHB, β-hydroxybutyrate; RAPA, rapamycin; p62, sequestosome-1; LC3, microtubule associated proteins 1A/1B light chain 3; CD11b, integrin alpha-M precursor; CD11a, integrin alpha-L precursor; CD18, integrin beta-2 precursor; SEM, standard error of the mean.

To investigate the relationship between BHB-induced increase in adhesion of neutrophils and impaired autophagy, the autophagy activator RAPA was used before BHB treatment. Compared with the control group, BHB treatment (1.6 m*M*, 2 h) increased (*P* < 0.01) the protein abundance of p62 while decreasing (*P* < 0.01) the ratio of protein abundance LC3 II to LC3 I in bovine neutrophils, both of which were reversed by RAPA treatment, a widely used activator of autophagy (**Figures 4A-C**). Compared with the control group, the protein abundance of membrane CD11a, CD11b, and CD18 was increased (*P* < 0.01) after BHB treatment, and RAPA reversed the above effects of BHB (*P* < 0.01, **Figures 4D-G**). Correspondingly, after BHB treatment, the number of adherent cells increased (*P* < 0.01) in the cell adhesion experiments, and the use of RAPA relives this effect (*P* < 0.01, **Figure 4H**).

### BHB inhibits the co-localization of CD11b and autophagosomes

**Figure 5:**
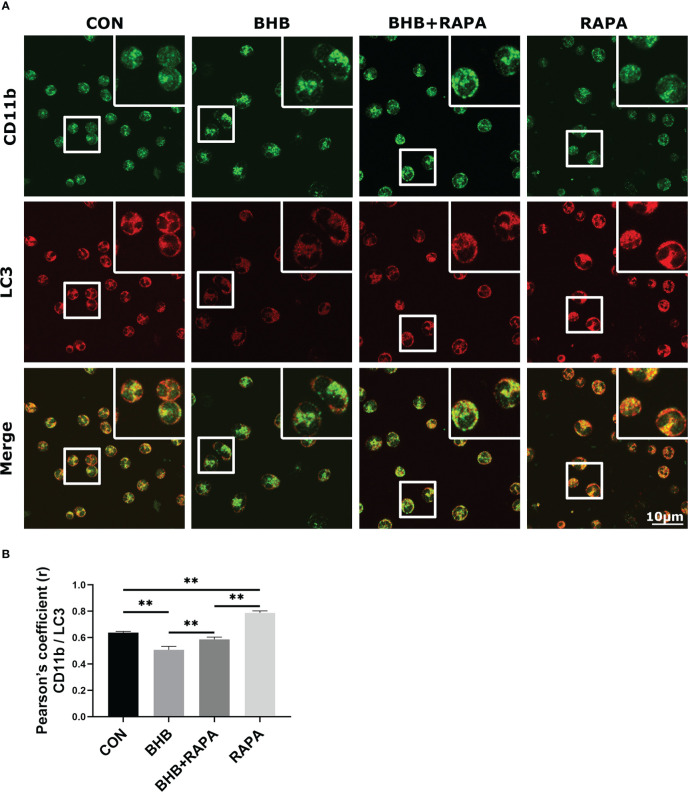
Role of autophagy in BHB induced depression of co-localization between CD11b and LC3. Rapamycin (100 n*M*, 2* h*) was used to activate autophagy. **(A)** Immunofluorescence data showed the co-localization of CD11b with LC3 in neutrophils. Magnification 400×. Scale bar = 10 μm. Nuclei/DAPI in blue, CD11b in green, and LC3 in red. Images here represent the typical common phenotype of each group. **(B)** Pearson’s correlation coefficient (r) between CD11b and LC3. Data were expressed as the mean ± SEM. A two-way analysis of variance (ANOVA) was performed to analyze differences between BHB and RARA, including a Bonferroni *post-hoc* analysis. Two-way ANOVA showed a significant difference in BHB × RAPA interaction (*P* < 0.01), including a Bonferroni *post-hoc* analysis. **P < 0.01 as statistically highly significant. CON, control; BHB, β-hydroxybutyrate; RAPA, rapamycin; LC3, microtubule associated proteins 1A/1B light chain 3; CD11b, integrin alpha-M precursor; SEM, standard error of the mean.

Compared with the control group, the co-localization between CD11b and LC3 was lower (*P* < 0.01) after BHB treatment (1.6 m*M*, 2 h). RAPA treatment enhanced their co-localization (*P* < 0.01) and relieved the effect of BHB (*P* < 0.01, **Figures 5A, B**). IF results further suggest that BHB can regulate the adhesion of neutrophils by inhibiting the circulation of adherent molecules.

### Autophagy is involved in BHB pro-inflammatory activation of bovine neutrophils

**Figure 6:**
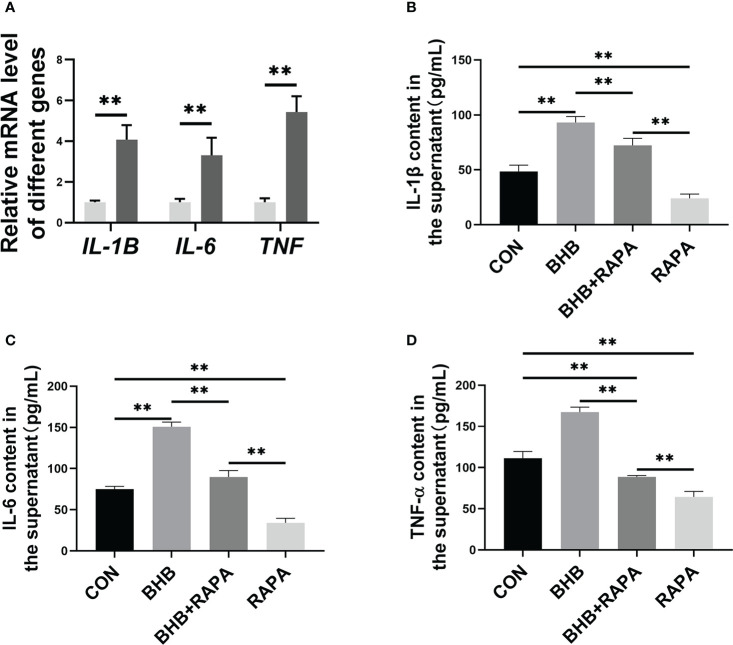
Role of autophagy in BHB promoting the production of pro-inflammatory factors in bovine neutrophils. Neutrophils were treated with 1.6 m*M* BHB for 2 h. **(A)** mRNA abundance of *IL-1B* and *IL-6*, and *TNF*. For qRT-PCR experiments, *β-Actin* and *YWHAZ* were used to normalize mRNA abundance. Data were expressed as the mean ± SEM. Data were analyzed with independent-sample t-tests. **(B-D)** Rapamycin (100 n*M*, 2* h*) was used to activate autophagy. The concentration of IL-1β, IL-6, and TNF-α in the supernatant of the medium. Data were expressed as the mean ± SEM. A two-way analysis of variance (ANOVA) was performed to analyze differences between BHB and RARA, including a Bonferroni *post-hoc* analysis. Two-way ANOVA showed a significant difference in BHB × RAPA interaction (*P* < 0.01), including a Bonferroni *post-hoc* analysis. **P < 0.01 as statistically highly significant. CON, control; BHB, β-hydroxybutyrate; RAPA, rapamycin; IL, Interleukin; TNF-α, Tumor necrosis factor-α; SEM, standard error of the mean.

Compared with the control group, BHB treatment significantly increased the mRNA abundance of *IL-1B*, *IL-6*, and *TNF* (*P* < 0.01, **Figure 6A**). Compared with the control group, the concentration of IL-1β, IL-6, and TNF-α in the supernatant was higher (*P* < 0.01) after BHB treatment (1.6 m*M*, 2 h), while RAPA reversed this effect (*P* < 0.01, **Figures 6B-D**). It’s consistent with our hypothesis that BHB inhibition of autophagy can participate in pro-inflammatory activation of bovine neutrophils.

## Discussion

Peripartum dairy cows often experience a state of negative energy balance (NEB). Severe NEB leads to fat mobilization, elevated blood BHB, and ketosis successively. SCK cows always have systemic inflammation characterized by increased pro-inflammatory cytokines including IL-1β, IL-6, and TNF-α, as well as increased positive acute phase response proteins HP and SAA in the blood (4). In our data, serum concentration of SAA, HP, CRP, TNF-α, IL-6, and IL-1β was all greater in cows with SCK, indicating the presence of systemic inflammation. Due to systemic inflammation, ketosis cows are more likely to experience mastitis, endometritis, and laminitis, which results in additional costs (24). BHB has been shown an important role in the regulation of inflammation. In bovine hepatocytes, BHB can promote the release of TNF-α, IL-6, and IL-1β by activating the NF-κB signaling pathway, thus aggravating liver damage (7). Additionally, BHB-induced oxidative stress activated the NF-κB signaling pathway and upregulated the release of pro-inflammatory factors in bovine endometrial cells (25). The present study not only confirmed the existence of systemic inflammation but also discovered a strong positive correlation between serum BHB and systemic inflammation.

Inflammation is a double-edged sword, which not only protects the body from infections but also leads to the dysfunction of cells and tissue damage when in excess. Adhesion between neutrophils and endothelial cells mediated by adhesion molecules is the first and key step of the inflammation process (26). Adhesion between neutrophils and endothelial cells promotes the transport of neutrophils to the inflammatory site and the release of inflammatory mediators, resulting in local and systemic inflammation and tissue damage (27). Importantly, the increase of the adhesion molecule CD11a has been shown to be closely associated with the release of pro-inflammatory factors and activation of neutrophil migration and phagocytosis, thereby regulating the inflammatory response (28). In human studies, hyperketonemia increases the expression of monocyte adhesion molecules CD11a and intercellular cell adhesion molecule 1 and promotes their adhesion to endothelial cells (16). Compared with healthy subjects, concentrations of circulating inflammatory markers and adhesion molecules were higher in diabetic patients with hyperketonemia (29). The above evidence suggests that the over-activation of neutrophil adhesion is highly related to the development of systemic inflammation. In cows, it was confirmed that the blood content of BHB was positively correlated with the incidence rate of inflammatory diseases such as mastitis and hysterias (3, 30). In the present study, BHB treatment increased the protein abundance of membrane adhesion molecules (CD11a, CD11b, and CD18), which was consistent with the enhanced adhesion function. These data suggested the elevated blood content of BHB, at least partially, is responsible for the systemic inflammation of SCK cows. The change in protein abundance is related to both gene transcription and protein degradation. In mice, BHB can regulate intracellular signal transduction and gene transcription by activating hydroxy-carboxylic acid receptor 2 (HCA2) (31). Additionally, HCA2 may be able to interfere with neutrophil adhesion to endothelial cells and with neutrophil migration in mice (32). Therefore, the BHB-induced increase in the abundance of adherent molecules may be caused by HCA2-mediated elevated transcription levels of these genes. However, in our data, the mRNA abundance of the above adhesion molecules was not affected significantly by BHB treatment. This suggests that the increased abundance of CD11a, b, and CD18 may be the result of the impaired degradation system in bovine neutrophils.

Autophagy is an important way of intracellular material turnover, maintains cell homeostasis, and helps their survival under stress (33). In both immune and non-immune cells, autophagy regulates cell adhesion by regulating endocytosis and the circulation of membrane integrin (17, 34). Inhibition of autophagy increases the adhesion of macrophages and promotes their migration to the renal in hyperglycemic mice (34). In Atg5 ^-/-^ mice, the infiltration of neutrophils in peripheral tissue increases (19). Autophagy associated adhesion strongly correlates to the metastasis of human breast cancer (35). The present study found that the autophagy of bovine neutrophils was inhibited after BHB treatment, along with the accumulation of CD11a, CD11b, and CD18. Importantly, activation of autophagy by RAPA reversed the effects of BHB enhancing adhesion, suggesting an underlying regulatory role of autophagy.

During the development of inflammation, adherent neutrophils produce inflammatory mediators such as IL-17, IL-6, and leukotriene B4. These substances induce endothelial cells, mesenchymal cells, and myeloid cells to release pro-inflammatory factors, including chemokines and matrix metalloproteinases, thereby expanding the inflammatory response and eventually leading to inflammatory injury (36). As an important participant in magnifying inflammation, the pro-inflammatory activation of neutrophils may be the driving factor of systemic inflammation in SCK cows. Some studies have shown that autophagy negatively regulates the pro-inflammatory activation in mouse macrophages (37), while it is still unclear in bovine neutrophils. In the present study, BHB treatment increased the mRNA abundance and the release of pro-inflammatory factors IL-1β, IL-6, and TNF-α of bovine neutrophils, while these effects were relieved when autophagy was active. Therefore, autophagy-mediated adhesion of blood neutrophils to vascular endothelial cells and the subsequent pro-inflammatory activation of neutrophils induced by a high concentration of blood BHB may contribute to the development of systemic inflammation in dairy cows with SCK. However, *in vivo* experimental data are needed to further confirm this view.

Extracellular vesicles (EVs), membrane-enclosed vesicles secreted by nearly all cell types, are enriched with cytokines, chemokines, and immunomodulatory factors which can participate in the modulation of immunity (38). They can transfer bioactive materials (e.g., proteins, nucleic acids, lipids, and metabolites) from host cells to both neighboring acceptor cells and distal recipient cells, thereby orchestrating a persistent communication network in extracellular spaces. Depending on the different host cell types and various bioactive contents, EVs can both have immunostimulatory and immunoinhibitory effects (39). In Lajqi’s report, they make a perfect presentation linking small EVs to the regulation of adaptive inflammatory features in innate immune cells (40). Their data show that small EVs may increase the production of pro-inflammatory mediators (e.g., TNF-α and IL-6) and boost transmigratory as well as phagocytic properties mediated by the TLR2/MyD88 pathway in murine neutrophils. Indeed, the increased release of EVs by neutrophils has been found to induce abundance of ICAM-1 on endothelial cells and promote cell adhesion in humans (41). Additionally, neutrophils also contain a releasable membrane-bound organelle named the secretory vesicle (also named granule) which can participate in immune modulation. In our previous study, it has been demonstrated that the enhanced release of the secretory vesicle by neutrophil degranulation can contribute to systemic inflammation. (23). Importantly, a variety of studies show that neutrophil tertiary granule is rich in Mac-1, which transfers to the cell membrane during degranulation through membrane fusion (42). Thus, the granules or EVs may be involved in regulation of BHB-induced neutrophil adhesion in cows with SCK and this needs further investigation in the future.

Continuous assembly and dismantling of adhesions at the cell front and tail produce the driving force for the forward movement of migrating cells (35). There is little research on the regulation mechanism of the turnover of focal adhesion. At the end of the 1980s, scientists first discovered the endocytosis and recycling of integrin, and subsequent studies confirmed that this cycle is related to autophagy (43). Starvation-induced autophagy leads to the degradation of integrins in the lysosomes, but this process is reversible as soon as cells are returned to conditions that inhibit autophagy (17). In the present study, the co-localization between CD11b and LC3 was confirmed, suggesting that autophagy may be also involved in the regulation of adhesion molecules turnover in bovine neutrophils. BHB treatment inhibited autophagy and decreased the co-localization between LC3 and CD11b in bovine neutrophils, while RAPA alleviated this effect. These data suggested that BHB may cause abnormal turnover of adhesion molecules by inhibiting autophagy, thereby leading to their increased membrane localization and finally regulating the adhesion function of bovine neutrophils.

In conclusion, the present study demonstrated that SCK cows exist systemic inflammation, which is related to a high concentration of BHB in blood. *In vitro* experiments, it is confirmed that pathological concentration of BHB promotes the adhesion of bovine neutrophils by inhibiting autophagy. Our data provide a new understanding of the development of systemic inflammation in SCK cows.

## Data availability statement

The original contributions presented in the study are included in the article/**Supplementary Material**. Further inquiries can be directed to the corresponding authors.

## Ethics statement

The animal study was reviewed and approved by Ethics Committee on the Use and Care of Animals of Jilin University.

## Author contributions

JH and KW wrote the manuscript and prepared all the figures. ML, DL, YL, LL, XD and SL participated in the experiment and the analysis of data. GL, and YS were involved in the study design and funding acquisition. ZM, TB, and WZ assume responsibility for oversight and leadership in the study. All authors contributed to the manuscript and approved the submitted version.
