# Supplementary material for: β-hydroxybutyrate enhances bovine neutrophil adhesion by inhibiting autophagy
Source: Front Immunol. 2023 Jan 11;13:1096813. doi: 10.3389/fimmu.2022.1096813 (PMC9874688; doi:10.3389/fimmu.2022.1096813)
Supplement: Supplementary file 1 [file Table_1.docx]

**Supplement Table 1.** The primers sequences used for quantitative real time-PCR.

| **Gene** | **Sequence number** | **Primer sequences (5’–3’)** | **Length (bp)** |
| --- | --- | --- | --- |
| *ITGAL* (CD11a) | NM_198221.2 | FOR: ACCCCACAAGTGACAACCTG | 286 |
|  |  | REV: TGAACAGCCGCAAACTGGTA |  |
| *ITGAM* (CD11b) | NM_001039957.1 | FOR: TCTCACAGCCAGCGGATCA | 241 |
|  |  | REV: GGCCTTTTACCACTGGGTCA |  |
| *ITGB2* (CD18) | NM_175781.1 | FOR: TGACGCTCTACCTGAGACCA | 208 |
|  |  | REV: ACGAAGGACCCGAAACCAAT |  |
| *SQSTM1* (p62) | NM_176641.1 | FOR: GGCCTACCTTCTGGGCAAG | 197 |
|  |  | REV: CCCCATCCTCATCGCGGTA |  |
| *MAP1LC3B* (LC3) | NM_001001169.1 | FOR: GCCGAACCTTCGAACAAAGA | 193 |
|  |  | REV: TTGAGCTGTAAGCGCCTTCT |  |
| *IL-1B* | NM_174093.1 | FOR: CCTTCATTGCCCAGGTTTCTG | 166 |
|  |  | REV: CCATCTCCCATGGAACCGAG |  |
| *IL-6* | NM_173923.2 | FOR: AATCTGGGTTCAATCAGGCGA | 199 |
|  |  | REV: GTGTTTGTGGCTGGAGTGGT |  |
| *TNF* | NM_173966.3 | FOR: CCCAGAGGGAAGAGCAGTCC | 195 |
|  |  | REV: TGTCTTCCAGCTTCACACCG |  |
| *YWHAZ* | NM_174814.2 | FOR: CACCTACTCCGGACACAGAAC | 200 |
|  |  | REV: TGACCTACGGGCTCCTACAA |  |
| *β-actin* | NM_173979.3 | FOR: CTAACAGTCCGCCTAGAAGCA | 402 |
|  |  | REV: GTCATCACCATCGGCAATGAG |  |

The corresponding protein name was added in the brackets after the gene symbol.
